# Antibiotic Minimal Selective Concentrations and Fitness Costs during Biofilm and Planktonic Growth

**DOI:** 10.1128/mbio.01447-22

**Published:** 2022-06-13

**Authors:** Karin Hjort, Elin Fermér, Po-Cheng Tang, Dan I. Andersson

**Affiliations:** a Department of Medical Biochemistry and Microbiology, Uppsala University, Uppsala, Sweden; McMaster University

**Keywords:** *Escherichia coli*, antibiotic resistance, biofilms, fitness, minimal selective concentration, planktonic

## Abstract

The use and misuse of antibiotics have resulted in the selection of difficult-to-treat resistant bacteria. Two key parameters that influence the selection of resistant bacteria are the minimal selective concentration (MSC) and the fitness cost of resistance, both of which have been measured during planktonic growth in several studies. However, bacterial growth most often occurs in biofilms, and it is unclear if and how these parameters differ under these two growth conditions. To address this knowledge gap, we compared a selection of several types of antibiotic-resistant Escherichia coli mutants during planktonic and biofilm growth to determine the fitness costs and MSCs. Biofilm-forming Escherichia coli strains are commonly found in catheter-associated and recurrent urinary tract infections. Isogenic strains of a biofilm-forming E. coli strain, differing only in the resistance mechanisms and the fluorescent markers, were constructed, and susceptible and resistant bacteria were grown in head-to-head competitions at various concentrations of antibiotics under planktonic and biofilm conditions. Mutants with resistance to five different antibiotics were studied. The results show that during both planktonic and biofilm growth, selection for the resistant mutants occurred for all antibiotics at sub-MICs far below the MIC of the antibiotic. Even though differences were seen, the MSC values and the fitness costs did not differ systematically between planktonic and biofilm growth, implying that despite the different growth modes, the basic selection parameters are similar. These findings highlight the risk that resistant mutants may, similarly to planktonic growth, also be selected at sub-MICs of antibiotics in biofilms.

## INTRODUCTION

Antibiotic resistance is a growing threat worldwide (1), and reports from the World Health Organization (WHO) (2) and the European Centre for Disease Prevention and Control (ECDC) (3) declare that the rapid increase in antibiotic resistance is one of the largest threats to public health globally. Historically, most of the research on antibiotic resistance was performed on bacteria growing planktonically, but the increasing awareness of biofilms being the major growth mode of bacteria during infections implies that more focus should be on understanding the selection of resistance during antibiotic exposure in biofilms. Biofilms are defined as matrix-enclosed bacterial populations adherent to each other and/or to surfaces or interfaces (4). The matrix is composed of extracellular polymeric substances and encases the cells in a protective layer (5). Bacterial biofilms are correlated with a wide range of infections, including but not limited to those linked to exogenous devices (6, 7) and chronic tissue infections (6). The infections are difficult to cure due to a wide range of molecular mechanisms that contribute to the high degree of antibiotic tolerance, such as impaired antibiotic diffusion in the extracellular matrix, altered physiology, heterogeneity, and reduced growth rates (8, 9), making biofilms more resilient to the effects of antibiotics and disinfectants (6). More specifically, Escherichia coli biofilms are the major contributor to recurrent urinary tract infections and important in causing indwelling medical device-related infections (10).

During the last decade, the knowledge that resistance selection and *de novo* resistance development occur not only above the MIC of the susceptible strain but also at subinhibitory concentrations has increased substantially (11–15). Thus, previous studies using different experimental setups have demonstrated the selection of resistant mutants at concentrations up to several hundredfold below the MIC of the susceptible strain (11, 12, 16). Gullberg et al. performed competition assays with isogenic pairs of susceptible and resistant strains that were allowed to compete for growth at a range of sub-MICs of antibiotics to determine the lowest antibiotic concentration that could enrich for resistant mutants (12). From this study, the concept of minimal selective concentration (MSC) was introduced and defined as concentrations above the MSC of an antibiotic that result in the enrichment of a resistant mutant over the susceptible strain in an otherwise isogenic population (15). Phrased another way, the MSC is the concentration of an antibiotic where the fitness cost of resistance is balanced by the antibiotic-conferred selection for the resistant mutant. Fitness cost, the reduction of relative fitness due to a resistance mechanism, influences the MSC (17, 18), and studies have shown that an increase in the fitness cost of a resistance mutation/gene results in, as expected, a corresponding increase in the MSC (12, 16). More recent studies including resistance selection in more complex microbial communities (18–25) have provided further support for the notion that sub-MICs of antibiotics can drive the selection of resistant mutants and that the low antibiotic concentrations found in many environments are now considered real threats with the potential to enrich for either preexisting or *de novo*-generated resistant pathogens (15). Also, subinhibitory concentrations of antibiotics have been shown to drive the shift from a planktonic to a biofilm growth mode across different species (26–31).

In this study, the selection and enrichment of resistant mutants of a biofilm-forming E. coli strain were investigated. By performing competitions between the susceptible wild type and resistant mutants at sub-MICs in a biofilm and during planktonic growth, we were able to compare the MSCs and fitness costs of resistance for these two bacterial lifestyles. Most importantly, our results demonstrate that the selection of resistant mutants was observed at sub-MICs of all antibiotics irrespective of the fitness cost of resistance and the growth conditions.

## RESULTS

### Experimental setup and rationale.

In this study, we used the clinically relevant uropathogenic biofilm-forming E. coli strain CFT073 to assess the MSCs and fitness costs for different antibiotics and resistant mutants. The choice of antibiotics examined was based on both the high clinical relevance of the antibiotic (trimethoprim, nitrofurantoin, and fosfomycin) for the treatment of E. coli infections and the presence of previous data on MSCs for comparative purposes between studies, e.g., trimethoprim (16, 23) and streptomycin (12, 16, 24). The choice of resistance mechanisms was based mainly on clinical relevance in E. coli (trimethoprim [*dfr* gene] [32], nitrofurantoin [*nfsAB* mutations] [33], and fosfomycin [*uhpT* mutation] [34]) as well as other bacteria (streptomycin [two different *rpsL* mutations] [35] and rifampicin [*rpoB* mutation] [36]).

**FIG 1:**
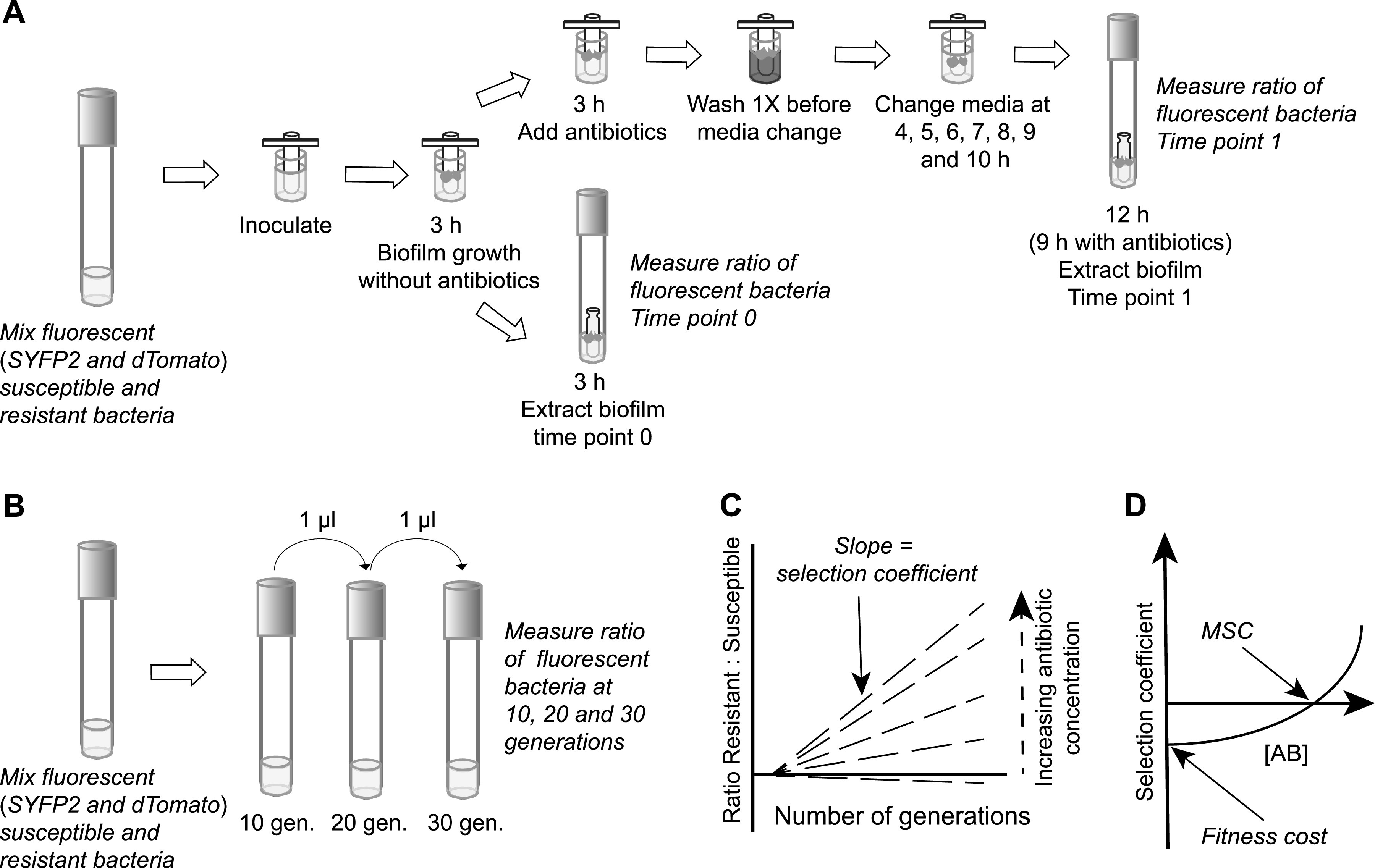
Schematic overview of the method to determine fitness costs and minimal selective concentrations (MSCs). Cultures of isogenic susceptible and resistant fluorescent strains were grown overnight separately and then mixed. (A) Fitness cost and MSC determinations during biofilm growth. FlexiPegs were inserted into a mix of fluorescent susceptible and resistant bacteria. After 3 h of incubation, the biofilm was extracted from a subset of the FlexiPegs to determine the initial ratio between susceptible and resistant strains before antibiotics were added (time point 0). The remaining FlexiPegs were transferred to different antibiotic concentrations and further incubated. To reduce the potential interference of planktonic cells, the biofilms on the FlexiPeg were briefly dipped into PBS every hour before they were transferred into fresh medium with or without antibiotics. After 9 h of incubation, the biofilm was harvested, and the ratios of susceptible to resistant fluorescently labeled bacteria were determined by flow cytometry at time points 0 and 1 (representing approximately 8 generations of biofilm growth). (B) Fitness cost and MSC determinations during planktonic growth. A mix of susceptible and resistant bacteria was transferred to different antibiotic concentrations for incubation, generating 10 generations of growth. Bacteria were analyzed by flow cytometry to determine the ratio between fluorescently labeled susceptible and resistant bacteria for each antibiotic concentration and time point (10, 20, and 30 generations). (C) Calculation of selection coefficients. The ratios of resistant to susceptible bacteria measured at different antibiotic concentrations were plotted over time to obtain the selection coefficients (slope of the curves). (D) Calculations of fitness costs and MSCs. The fitness costs and MSCs were estimated by plotting the selection coefficient (panel C) as a function of the antibiotic concentration (AB). The intercept with the *x* axis is the MSC value, and the intercept with the *y* axis is the fitness cost.

We performed competition experiments between susceptible and resistant bacteria genetically tagged with different fluorescent markers at subinhibitory antibiotic concentrations in rich growth media (see Fig. 1A to D for a schematic outline of the experiment) (12, 16). During biofilm growth (Fig. 1A), ratios were obtained after 8 generations of growth on FlexiPegs, an in-house-modified Calgary device for biofilm growth (37). The mixes of susceptible and resistant bacteria were allowed to attach to the FlexiPegs for 3 h in the absence of antibiotics, and antibiotics were then added. After an additional 9 h of incubation with several medium changes (to reduce any potential contribution of planktonic free cells to biofilm growth), the bacteria were removed from the FlexiPegs by vortexing, and the ratio of susceptible to resistant bacteria was measured by flow cytometry (see Fig. S1 in the supplemental material). Control experiments showed that the planktonic cells present in the growth medium in the microwells where the FlexiPegs were incubated had a minimal impact on biofilm growth (see Fig. S2 in the supplemental material). Thus, when the growth of the biofilm with the continuous presence of planktonic cells in the microwells was compared with that after several growth medium changes 3, 4, 5, 6, 7, 8, 9, and 10 h after inoculation (Fig. 1A), the numbers of CFU per FlexiPeg were similar at 3, 6, and 12 h (see Fig. S2 in the supplemental material), indicating that there was little to no reattachment of planktonic bacteria to the biofilm during this time period. For planktonic growth, ratios of susceptible to resistant bacteria (see Fig. S3 in the supplemental material) were measured over 30 generations of growth (Fig. 1B) by flow cytometry, as previously described (12, 16).

10.1128/mbio.01447-22.1 FIG S1 Competition experiments in biofilms between an isogenic pair of susceptible and resistant E. coli strains at different concentrations of antibiotics. Plotted are the ratios of resistant to susceptible bacteria at different antibiotic concentrations as a function of time. The data are based on the means from three biological replicates for each experiment and a dye swap, for six samples per antibiotic concentration in total. (A) Ratios of trimethoprim-resistant to -susceptible E. coli strains (*bglGFB*::*dfr SYFP2* [DA71530] versus wild-type [wt] *dTomato* [DA58420] and *bglGFB*::*dfr dTomato* [DA71531] versus wt *SYFP2* [DA58419]) at different antibiotic concentrations as a function of time. (B) Ratios of nitrofurantoin-resistant to -susceptible E. coli strains (Δ*nfsAB SYFP2* [DA69418] versus wt *dTomato* [DA58420] and Δ*nfsAB dTomato* [DA69419] versus wt *SYFP2* [DA58419]) at different antibiotic concentrations as a function of time. (C) Ratios of fosfomycin-resistant to -susceptible E. coli strains (*uhpT* STOP 5 aa *SYFP2* [DA66040] versus wt *dTomato* [DA58420] and *uhpT* STOP 5 aa *dTomato* [DA66041] versus wt *SYFP2* [DA58419]) at different antibiotic concentrations as a function of time. (D) Ratios of streptomycin-resistant to -susceptible E. coli strains (*rpsL* K42R *SYFP2* [DA71952] versus wt *dTomato* [DA58420] and *rpsL* K42R *dTomato* [DA71953] versus wt *SYFP2* [DA58419]) at different antibiotic concentrations as a function of time. (E) Ratios of streptomycin-resistant to -susceptible E. coli strains (*rpsL* K42N *SYFP2* [DA66038] versus wt *dTomato* [DA58420] and *rpsL* K42N *dTomato* [DA66039] versus wt *SYFP2* [DA58419]) at different antibiotic concentrations as a function of time. (F) Ratios of rifampicin-resistant to -susceptible E. coli strains (*rpoB* S531L *SYFP2* [DA66034] versus wt *dTomato* [DA58420] and *rpoB* S531L *dTomato* [DA66035] versus wt *SYFP2* [DA58419]) at different antibiotic concentrations as a function of time. Download FIG S1, EPS file, 0.2 MB. Copyright © 2022 Hjort et al. 2022 Hjort et al. https://creativecommons.org/licenses/by/4.0/ This content is distributed under the terms of the Creative Commons Attribution 4.0 International license.

10.1128/mbio.01447-22.2 FIG S2 Cell growth on FlexiPeg. E. coli growth on FlexiPeg (26) was analyzed as CFU per peg (2, 4, 6, and 12 h after inoculation with 2 × 10^4^ to 5 × 10^4^ cells per well) with (□) or without (○) intermittent medium changes at 3, 4, 5, 6, 7, 8, 9, and 10 h (discarding planktonic cells) or with additions of 6 mg/L streptomycin (□■). Two competition experiments with fluorescently labeled (*SYFP2* and *dTomato*) isogenic streptomycin-susceptible and -resistant strains (*rpsL* K42N *SYFP2* [DA66038] versus wt *dTomato* [DA58420] and *rpsL* K42N *dTomato* [DA66039] versus wt *SYFP2* [DA58419]) were conducted with (■) or without (□) the addition of 6 mg/L of streptomycin (MIC of 12 mg/L in brain heart infusion medium). The mean values with standard errors of the means are based on the results from four to six biological replicates and a dye swap for the streptomycin competition experiment. Download FIG S2, EPS file, 0.08 MB. Copyright © 2022 Hjort et al. 2022 Hjort et al. https://creativecommons.org/licenses/by/4.0/ This content is distributed under the terms of the Creative Commons Attribution 4.0 International license.

10.1128/mbio.01447-22.3 FIG S3 Competition experiment during planktonic growth between an isogenic pair of susceptible and resistant E. coli strains at different concentrations of antibiotics. Plotted are the ratios of resistant to susceptible bacteria at different antibiotic concentrations as a function of time. The data are based on the means from two independent experiments with four biological replicates for each experiment and a dye swap, for 16 samples in total per antibiotic concentration. (A) Ratios of trimethoprim-resistant to -susceptible E. coli strains (*bglGFB*::*dfr SYFP2* [DA71530] versus wt *dTomato* [DA58420] and *bglGFB*::*dfr dTomato* [DA71531] versus wt *SYFP2* [DA58419]) at different antibiotic concentrations as a function of time. (B) Ratios of nitrofurantoin-resistant to -susceptible E. coli strains (Δ*nfsAB SYFP2* [DA69418] versus wt *dTomato* [DA58420] and Δ*nfsAB dTomato* [DA69419] versus wt *SYFP2* [DA58419]) at different antibiotic concentrations as a function of time. (C) Ratios of fosfomycin-resistant to -susceptible E. coli strains (*uhpT* STOP 5 aa *SYFP2* [DA66040] versus wt *dTomato* [DA58420] and *uhpT* STOP 5 aa *dTomato* [DA69420] versus wt *SYFP2* [DA58419]) at different antibiotic concentrations as a function of time. (D) Ratios of streptomycin-resistant to -susceptible E. coli strains (*rpsL* K42R *SYFP2* [DA71952] versus wt *dTomato* [DA58420] and *rpsL* K42R *dTomato* [DA71953] versus wt *SYFP2* [DA58419]) at different antibiotic concentrations as a function of time. (E) Ratios of streptomycin-resistant to -susceptible E. coli strains (*rpsL* K42N *SYFP2* [DA66038] versus wt *dTomato* [DA58420] and *rpsL* K42N *dTomato* [DA66039] versus wt *SYFP2* [DA58419]) at different antibiotic concentrations as a function of time. (F) Ratios of rifampicin-resistant to -susceptible E. coli strains (*rpoB* S531L *SYFP* [DA66034] versus wt *dTomato* [DA58420] and *rpoB* S531L *dTomato* [DA66035] versus wt *SYFP2* [DA58419]) at different antibiotic concentrations as a function of time. Download FIG S3, EPS file, 0.9 MB. Copyright © 2022 Hjort et al. 2022 Hjort et al. https://creativecommons.org/licenses/by/4.0/ This content is distributed under the terms of the Creative Commons Attribution 4.0 International license.

For both planktonic and biofilm growth, the obtained ratios of resistant to susceptible bacteria during growth in the presence of different antibiotic concentrations were then plotted over time to obtain the selection coefficients, i.e., the slope of the curves (Fig. 1C). These calculated selection coefficients were then plotted against the antibiotic concentrations to estimate the minimal selective concentration (intercept on the *x* axis) and the fitness cost (intercept on the *y* axis) (Fig. 1D) (12). All competition experiments were performed with fluorescent dye swaps between the resistant and susceptible bacteria to correct for any potential effect of the fluorescent markers on growth and competitive ability. In total, 16 and 6 independent competition experiments were performed for planktonic growth and biofilm conditions, respectively. No measurable difference in fitness cost was observed for the two different fluorescent markers *SYFP2* and *dTomato* during biofilm or planktonic growth (see Fig. S4 in the supplemental material).

10.1128/mbio.01447-22.4 FIG S4 Fitness cost measurements of the fluorescent tags during planktonic and biofilm growth. (A) Planktonic competition experiment with two fluorescently tagged E. coli CFT073 strains (*galK*::*kan-J23101-SYFP2* [DA58419] and *galK*::*kan*-*J23101-dTomato* [DA58420]), which were competed for 144 h (approximately 60 generations). The cell cultures were diluted every day by transferring 1 μL to 1 mL of fresh medium (incubated in shaking tubes). At 48, 96, and 144 h, the cells were enumerated by flow cytometry, and the ratios between them (six biological replicates) were plotted against time. (B) Biofilm competition experiment with two fluorescently tagged E. coli CFT073 strains (*galK*::*kan-J23101-SYFP2* [DA58419] and *galK*::*kan*-*J23101-dTomato* [DA58420]), which were competed for 144 h (approximately 48 generations). At 48, 96, and 144 h of incubation, the biofilm was harvested, and the cells were enumerated by flow cytometry. The ratios between the two fluorescently labeled E. coli isolates (eight biological replicates) were plotted against time. Download FIG S4, EPS file, 0.9 MB. Copyright © 2022 Hjort et al. 2022 Hjort et al. https://creativecommons.org/licenses/by/4.0/ This content is distributed under the terms of the Creative Commons Attribution 4.0 International license.

### Fitness costs of antibiotic resistance during planktonic and biofilm growth.

**FIG 2:**
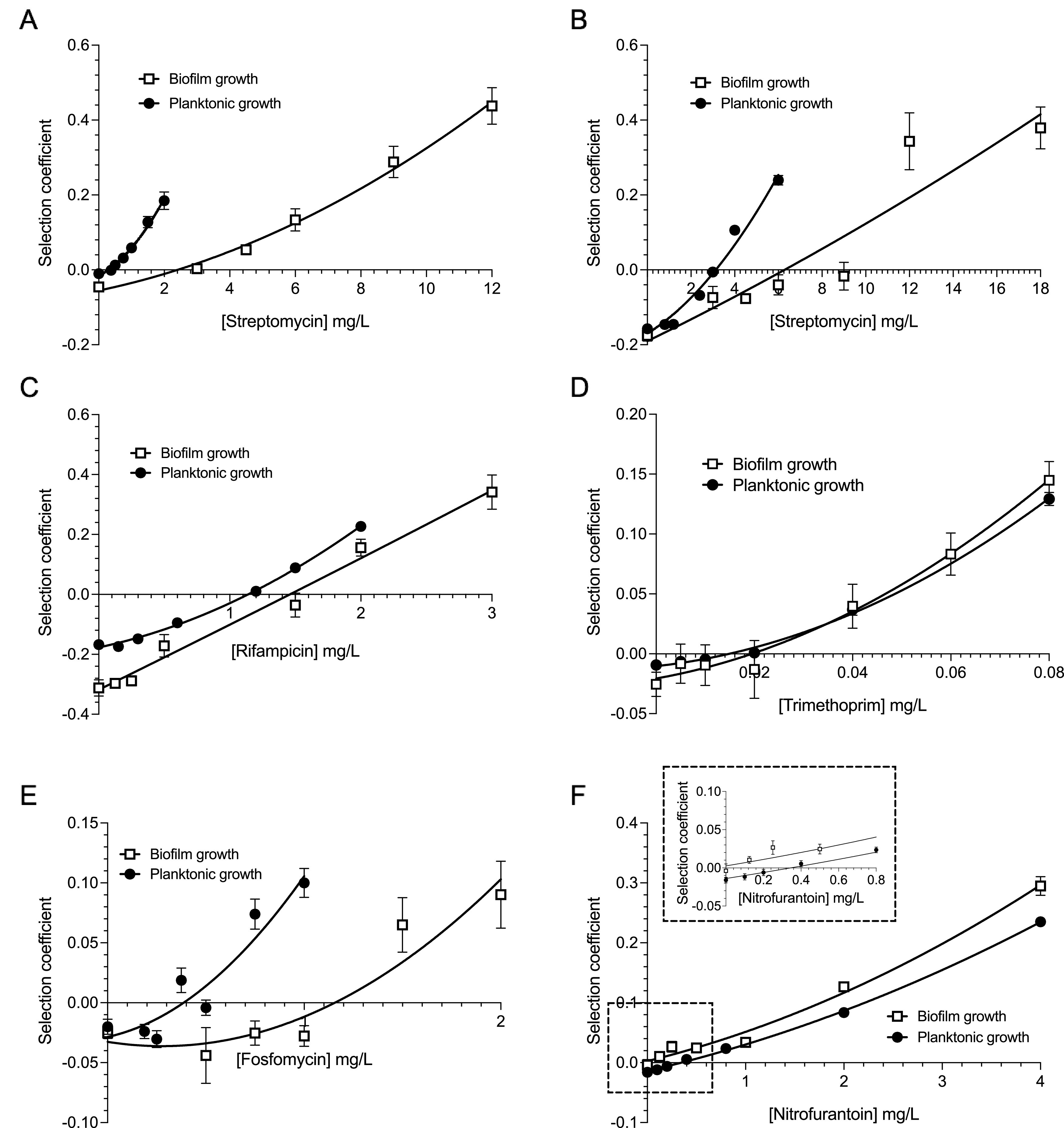
Determination of fitness costs and MSCs for different resistant mutants and antibiotics. Selection coefficients obtained from competitions between susceptible and resistant bacteria (see Fig. S1 and S3 in the supplemental material) were plotted as a function of the antibiotic concentration for planktonic and biofilm growth. Standard errors of the means are from two independent experiments with four biological replicates and a dye swap, for 16 samples in total for planktonic growth, and with three biological replicates and a dye swap, for 6 samples in total for biofilm growth. Resistant mutants were E. coli *rpsL* K42R (A), E. coli *rpsL* K42N (B), E. coli *rpoB* S531L (C), E. coli *bgl*::*dfr* (D), E. coli *uhpT* STOP 5 aa (E), and E. coli Δ*nfsAB* (F).

The fitness cost determinations are based on measuring the bacterial growth reduction displayed by the resistant mutants in the absence of antibiotics. As described above, the fitness cost is obtained from competitions between susceptible and resistant strains at different antibiotic concentrations and a plot of the selection coefficients as a function of antibiotic concentrations where the intercept on the *y* axis (antibiotic concentration = 0) defines the fitness cost (Fig. 2). Depending on the resistance mechanisms and growth conditions, the fitness cost for planktonic growth varied from 1% for the trimethoprim (*bgl*::*dfr*)-resistant mutant to 18% for the rifampicin (*rpoB* S531L)-resistant mutant, and that during biofilm growth fitness cost varied from 2.8% for the fosfomycin (*uhpT* STOP 5 aa (the fifth amino acid was changed to a stop codon, TTA))-resistant mutant up to 37.4% for the rifampicin (*rpoB* S531L)-resistant mutant (Table 1). When comparing the six different resistant mutants, the fitness costs were slightly higher for biofilm than for planktonic growth for three of the mutants (Fig. 2B to D and Table 1). Thus, for *rpsL* K42R (streptomycin resistance), *rpoB* S531L (rifampicin resistance), and *bgl*::*dfr* (trimethoprim resistance), the fitness cost was 2- to 4-fold higher in biofilms. However, the increase in the fitness cost for growth in the biofilm was highly significant (*P* < 0.0001 by a two-tailed *t* test) for streptomycin (*rpsL* K42R) and rifampicin (*rpoB* S531L) resistance. For *rpsL* K42N (streptomycin resistance) and fosfomycin (*uhpT* STOP 5 aa), the costs were similar for both biofilm and planktonic growth (Fig. 2B and E and Table 1). For the nitrofurantoin (Δ*nfsAB*)-resistant mutant, no fitness cost could be estimated during biofilm growth, whereas during planktonic growth, it was 1.4% (Fig. 2F and Table 1). In conclusion, the fitness costs were similar (less than 2-fold in most cases) when comparing growth in biofilms and planktonic culture.

**TABLE 1:** MICs, minimal selective concentrations, and fitness costs for resistant mutants during planktonic and biofilm growth^*a*^

Resistance	Resistance mutation	Planktonic growth						Biofilm growth					
		MIC mg/L for susceptible strain	Fitness cost (%)	SEM of fitness cost (%)	MSC (mg/L)	SEM of MSC (mg/L)	MIC/MSC ratio	MBIC mg/L for susceptible strain	Fitness cost (%)	SEM of fitness cost (%)	MSC (mg/L)	SEM of MSC (mg/L)	MBIC/MSC ratio
Streptomycin	*rpsL* K42N	48	17.8	0.5	3.1	0.06	15	96	22	2.5	5.9	0.6	15
Streptomycin	*rpsL* K42R	48	2.4	0.5	0.3	0.03	160	96	9.6	0.8	2.2	0.2	44
Rifampicin	*rpoB* S531L	24	18	0.8	1.1	0.03	22	>384	37.4	1.9	1.5	0.08	>256
Nitrofurantoin	Δ*nfsAB*	32	1.4	0.4	0.4	0.08	80	256	ND		ND		ND
Trimethoprim	*bgl*::*dfr*	1	1	0.3	0.017	0.003	50	>512	2.9	0.9	0.023	0.008	>25,000
Fosfomycin	*uhpT* STOP 5 aa	32	2.9	0.7	0.4	0.06	80	512	2.8	0.7	1.2	0.1	ND

a ND, the fitness cost or MSC value could not be determined; MBIC, minimal biofilm inhibitory concentration measured after 24 h; MSC, minimal selective concentration; MIC, minimal inhibitory concentration; SEM, standard error of the mean.

### Minimal selective concentrations during planktonic and biofilm growth.

**FIG 3:**
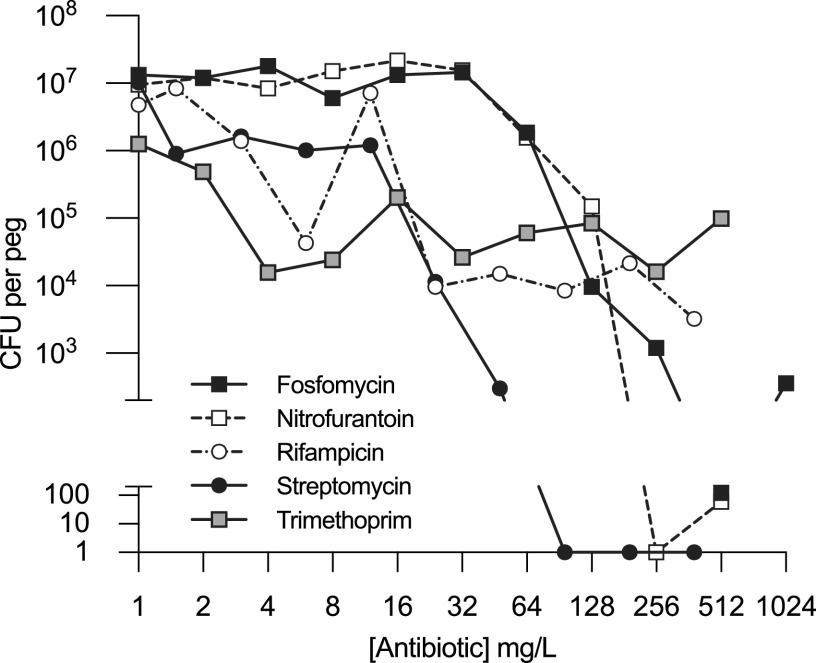
Antibiotic tolerance measured as the minimal biofilm inhibitory concentration (MBIC). The MBIC was determined after 24 h of biofilm formation in brain heart infusion medium. The biofilm was pregrown for 24 h before antibiotics were added. The biofilms were harvested after an additional 24 h of incubation with antibiotic exposure. Data are the medians from 4 to 8 biological replicates.

To better understand the impact of subinhibitory antibiotic concentrations on the selection of antibiotic resistance, the minimal selective concentration (MSC) was determined during both biofilm and planktonic growth. As described above, the MSC is obtained from competitions between susceptible and resistant strains at different antibiotic concentrations and from a plot of the selection coefficients as a function of the antibiotic concentration where the intercept on the *x* axis defines the MSC (Fig. 2). The difference in MSCs for the streptomycin-resistant mutants depended on the amino acid substituted in the *rpsL* gene, with a 2-fold increase (*P* = 0.05 by a two-tailed *t* test; *t* = 4.747; df = 5.116) in the MSC during biofilm versus planktonic growth for *rpsL* K42N and a 7-fold increase (*P* = 0.0003 by a two-tailed *t* test; *t* = 8.596; df = 5.192) for *rpsL* K42R (Fig. 2A and B and Table 1). For fosfomycin (*uhpT* STOP 5 aa) the increase in the MSC was 3-fold (*P* = 0.0085 by a two-tailed *t* test; *t* = 5.080; df = 3.739) for biofilm compared to planktonic growth, and for the rifampicin (*rpoB* S531L)- and trimethoprim (*bgl*::*dfr*)-resistant mutants, the MSCs were similar or the same independent of growth (Fig. 2C to E and Table 1), whereas for nitrofurantoin (Δ*nfsAB*), the MSC values could not be determined in the biofilm since these resistances showed no measurable fitness cost, and therefore, no intercept on the *x* axis was obtained (Fig. 2F and Table 1). To allow a comparison of how much lower the MSCs were than the MICs, we determined the MIC values during planktonic growth by broth microdilution and during biofilm growth by minimal biofilm inhibitory concentrations (MBICs) after 24 h of biofilm formation (Fig. 3). As expected, the biofilm MBIC values were significantly higher for all resistant mutants than MIC for planktonic growth, varying between a 2-fold-higher value for the streptomycin-resistant mutants to a >512-fold-higher value for trimethoprim (Table 2). The large difference between MBIC and MIC values leads to a drastic difference between MBIC/MSC and MIC/MSC ratios for rifampicin (>256 versus 22) and trimethoprim (50 versus >25,000) (Table 1). In conclusion, these results show that for all antibiotics, the resistant mutants were selected at subinhibitory concentrations during planktonic as well as biofilm growth.

**TABLE 2:** MICs and minimal biofilm inhibitory concentrations for resistant mutants^*a*^

DA no.	Genotype	Streptomycin		Rifampicin		Nitrofurantoin		Trimethoprim		Fosfomycin	
		MIC mg/L (BMD) in BHI	MBIC mg/L 24 h in BHI	MIC mg/L (BMD) in BHI	MBIC mg/L 24 h in BHI	MIC mg/L (BMD) in BHI	MBIC mg/L 24 h in BHI	MIC mg/L (BMD) in BHI	MBIC mg/L 24 h in BHI	MIC mg/L (BMD) in BHI	MBIC mg/L 24 h in BHI
DA58419	*galK*::*kan-J23101-SYFP2*	48	96	24	>384	32	256	1	>512	32	512
DA58420	*galK*::*kan-J23101-dTomato*	48	96	24	>384	32	256	1	>512	32	512
DA66038	*galK*::*kan-J23101-SYFP2 rpsL* K42N	>1,536	ND								
DA66039	*galK*::*kan-J23101-dTomato rpsL* K42N	>1,536	ND								
DA71952	*galK*::*kan-J23101-SYFP2 rpsL* K42R	>1,536	ND								
DA71953	*galK*::*kan-J23101-dTomato rpsL* K42R	>1,536	ND								
DA66034	*galK*::*kan-J23101-SYFP2 rpoB* S531L			>384	ND						
DA66035	*galK*::*kan-J23101-dTomato rpoB* S531L			>384	ND						
DA69418	*galK*::*kan-J23101-SYFP2* Δ*nfsAB*					128	ND				
DA69419	*galK*::*kan-J23101-dTomato* Δ*nfsAB*					128	ND				
DA71530	*galK*::*kan-J23101-SYFP2 bgl*::*dfr*							>2,048	ND		
DA71531	*galK*::*kan-J23101-dTomato bgl*::*dfr*							>2,048	ND		
DA66040	*galK*::*kan-J23101-SYFP2 uhpT* STOP 5 aa									1,024	ND
DA66041	*galK*::*kan-J23101-dTomato uhpT* STOP 5 aa									1,024	ND

a BMD, broth microdilution; MBIC, minimal biofilm inhibitory concentration; BHI, brain heart infusion medium; ND, not determined; MIC, minimal inhibitory concentration.

## DISCUSSION

In this study, we used the biofilm-forming E. coli CFT073 strain (37–39) to study the selection of resistant mutants at subinhibitory concentrations of antibiotics under planktonic and biofilm growth conditions. The antibiotics included were streptomycin and rifampicin together with antibiotics that are clinically relevant for the treatment of E. coli infections, such as trimethoprim, nitrofurantoin, and fosfomycin. The antibiotic resistance mechanisms for nitrofurantoin (Δ*nfsAB*), fosfomycin (*uhpT*), and trimethoprim (*dfr*) used in our study are common among clinical isolates of E. coli and also identified as resistance mutations in *in vitro* selections (32–34).

Fitness cost in the form of a reduction in the growth rate is well characterized for antibiotic resistance mutations in the absence of antibiotics (17, 40) both during planktonic growth in the laboratory and in animal models (41, 42). Fitness cost measurements in biofilms are limited, and to the best of our knowledge, there has been only one previous study published (43). Santos-Lopez et al. showed that ciprofloxacin-resistant mutants evolving under biofilm conditions were more fit relative to their parental strain than resistance mutants that evolved planktonically (43). Our mutations were not evolved under different conditions, but instead, we compared the fitness costs of the same mutant under both biofilm and planktonic conditions. The fitness cost during planktonic growth measured in our experiments for *rpsL* K42N, *rpsL* K42R, *rpoB* S531L, and Δ*nfsAB* in E. coli CFT073 was in agreement with previous measurements (33, 36, 44), whereas for the *uhpT* STOP 5 aa mutant, the cost was lower in this study than in a previous report (34). The reason for this is unclear but might be due to the selection of compensatory mutations during the growth and handling of the resistant strain. In biofilms, most of the resistant mutants showed a fitness cost similar to that for planktonic growth, with a maximum of a 4-fold increase displayed by the streptomycin-resistant mutant *rpsL* K42R (Table 1).

These findings are to some extent opposite of what has been observed in other biofilm models (45, 46). They show that the complex environment that a biofilm provides with niche differentiation and versatile growth rates decreases the impact of the fitness cost on selection (45, 46). According to Ahmed et al., a strain with a high fitness cost ciprofloxacin resistance mutation was not outcompeted by the susceptible strain; instead, a small subpopulation of the resistant strain remained in a colony biofilm model (45). The window where most of the growth takes place in a biofilm and where the fitness cost is most important is in the attachment and growth (cell division) phases. As the biofilm matures, the rate of growth decreases, leading to general antibiotic tolerance (47). Similar results were obtained in a biofilm flow cell study where resistant mutants of E. coli present in an established biofilm did not substantially decrease after antibiotic selection was removed despite having a high fitness cost (46). However, these models take into account all stages of biofilms, including a mature biofilm. Thus, the differences between our study and previous work can be explained by the fact that we examine the early phases of biofilm formation and previous work examined later stages.

Our results show that resistance selection takes place at subinhibitory concentrations for all mutants during planktonic and biofilm growth (see Table 3 for a compilation and comparison of the present and previous studies). It is well established for planktonic growth that the selection of antibiotic-resistant mutants takes place below the MIC (11–13), which was also the case for the antibiotic-resistant mutants studied in our experiment. It is also notable for the mutants studied here that even though the MIC values are generally higher in biofilms than those under planktonic conditions, the MSC values are relatively similar. While our focus was on the determination of MSCs in defined biofilms for comparison to planktonic growth, other studies have studied selection below the MIC in complex biofilm and planktonic environments containing many different bacterial species (19–25). In these studies, selection of resistance to tetracycline, ciprofloxacin, streptomycin, trimethoprim, erythromycin, cefotaxime, gentamicin, kanamycin, and oxytetracycline occurred at subinhibitory concentrations. The MSCs that we obtained here for fosfomycin, rifampicin, and nitrofurantoin are the first to be determined for biofilm and planktonic growth. In the case of nitrofurantoin, no MSC value could be determined in the biofilm due to the low/no fitness cost of the particular mutant used (Fig. 2F). For rifampicin, an MSC was observed for biofilm growth that was similar to that for planktonic growth. For streptomycin, we used two resistant mutants with different fitness costs during planktonic (2.4% for *rpsL* K42R and 17.8% for *rpsL* K42N) and biofilm (9.6% for *rpsL* K42R and 22% for *rpsL* K42N) growth (Table 1). The MSC for the *rpsL* K42R mutant was 0.3 mg/L (160-fold lower than the MIC) during planktonic growth, which is slightly lower than the MSC determined previously for the same mutation in Salmonella enterica serovar Typhimurium (Table 3) (12). The MSC during biofilm growth (2.2 mg/L) was higher than that for planktonic growth but well below the MBIC (40-fold), and it is in line with published data on selection for streptomycin-resistant heterotrophic bacteria in a wastewater biofilm (fiber ball) treatment system (Table 3) (24).

**TABLE 3:** Comparison of minimal selective concentrations determined under different growth conditions and in different experimental systems

Antibiotic	Exptl setup	Gene location	Gene(s) analyzed	Planktonic MSC	Biofilm MSC	Reference
Trimethoprim	Competition, E. coli	Chromosomal	*dfr* (pUUH 239)	17 μg/L	23 μg/L	This study
	Competition, E. coli	Chromosomal	*dfr* (pUUH 239)	<2 μg/L		16
	Competition, E. coli	Plasmid	*dfr* (pUUH 239)	33 μg/L		16
	Complex bacterial community, different methods of measurement		*intI1* copy no.	31.25–250 μg/L		20
	Competition, E. coli	Plasmid (pMK)	*dfrA1*, *dfrA5*, *dfrA12*, *dfrA17*	42–50 μg/L		23
	Biofilm, complex E. coli community		Trimethoprim-resistant E. coli		10–100 μg/L^*a*^	23

Streptomycin	Competition, E. coli	Chromosomal	*rpsL* K42N	3.1 mg/L	5.9 mg/L	This study
	Competition, E. coli	Chromosomal	*rpsL* K42R	0.3 mg/L	2.4 mg/L	This study
	Competition, *S.* Typhimurium	Chromosomal	*rpsL* K42R	1 mg/L		12
	Biofilm, complex bacterial community	Metagenome	Aminoglycoside resistance genes		0.1–1 mg/L^*a*^	24
	Biofilm, complex bacterial community		Streptomycin-resistant bacteria		1–5 mg/L^*a*^	24

Gentamicin	Competition, E. coli	Chromosome	*aacC1*	0.2 mg/L		18
	Competition, E. coli with complex microbial community	Chromosome	*aacC1*	10 mg/L		18

Kanamycin	Competition, E. coli	Chromosome	*aph(3*′*)-IIb*	0.5 mg/L		18
	Competition, E. coli with complex microbial community	Chromosome	*aph(3*′*)-IIb*	7 mg/L		18

Tetracycline	Competition, *S.* Typhimurium	Chromosomal	Tn*10*dTet	15 μg/L		12
	Biofilm, complex bacterial community		Tetracycline-resistant bacteria		1–10 μg/L^*a*^	25
	Biofilm, complex bacterial community		Metagenome, qPCR *tetA* and *tetG*		1 μg/L	25

Ciprofloxacin	Competition, E. coli	Chromosomal	*gyrA* S83L	0.1 μg/L		12
	Competition, E. coli	Chromosomal	*gyrA* D87N	2.5 μg/L		12
	Complex community		Ciprofloxacin-resistant bacteria	1–5 μg/L^*a*^		22
	Complex E. coli community		Ciprofloxacin-resistant E. coli	1–5 μg/L^*a*^		22
	Biofilm, complex E. coli community		Ciprofloxacin-resistant E. coli		1–10 μg/L^*a*^	22
	Biofilm, complex E. coli community	Metagenome	*parC* (S80 and E84) and *gyrA* (S83 and D87)		1–10 μg/L^*a*^	22

a NOEC (no-observed-effect concentration) to LOEC (lowest-observed-effect concentration); MSC, minimal selective concentration.

The planktonic MSC of trimethoprim was 17 μg/L, which was between previously reported MSC values for the same *dfr* gene (pUUH239.2 plasmid derived) (48) located on the chromosome in E. coli (MG1655) (16) and the MSCs of four different *dfr* genes present in E. coli measured by Kraupner et al. (23). The higher MSC value measured by Kraupner et al. is probably due to the higher fitness cost (>50%) associated with the plasmid location of the *dfr* genes. An increase in the fitness cost was also observed previously for the *dfr* gene when present in its original location on the pUUH239.2 plasmid (Table 3) (16). The MSC value (*dfr*) determined for biofilm growth (23 μg/L) in this study was similar to that for planktonic growth, and it was also in the same range as the MSC determined previously in a complex community of an E. coli biofilm (23). The high tolerance (MBIC/MSC ratio of >25,000) against trimethoprim in a biofilm combined with the low fitness cost for the plasmid-located *dfr* gene (Table 1) might suggest that this resistance could be easily acquired by horizontal gene transfer (HGT) in clinical settings.

In conclusion, the data presented here suggest that both fitness costs and minimal selective concentrations are relatively similar when comparing the same resistant mutants and antibiotics under two different bacterial lifestyles. However, when comparing results from different studies, it is clear that the specific antibiotic, the type of resistance mechanism, and the complexity of the microbial community examined influence fitness costs and MSCs in as-yet-unpredictable ways. Of special importance is the impact of microbiological complexity (the presence of several bacterial species, predators, and complex nutrient conditions, etc.) on selection and MSCs, where it has been suggested that MSCs are higher in complex communities because the free concentration of a drug is reduced and the cost of resistance is higher, resulting in an apparent increase in the MSC (18). However, from the limited number of studies that allow comparisons of single species and the community, this notion is not fully supported. Thus, for tetracycline, the MSC is lower in the community (1.5- to 15-fold), depending on whether resistance is measured by the presence of resistant bacteria or by quantitative PCR (qPCR) (*tetGA* genes), than for single species (1 to 10 μg/L versus 15 μg/L) (12, 25). For ciprofloxacin and gentamicin/kanamycin, the opposite is observed, with the MSC being higher in the community than for single species for ciprofloxacin (1 to 10 μg/L versus 0.1 to 2.5 μg/L) (12, 22) and kanamycin/gentamicin (1 mg/L versus 0.025 mg/L) (18). These findings accentuate the need for further comparative studies of these key parameters in different environments and with different types of resistance mechanisms and antibiotics to better understand how, when, and where resistant bacteria are enriched as a result of antibiotic exposure (49). Finally, from a clinical perspective, the low MSCs observed during biofilm growth suggest that even if the concentrations of some antibiotics are reduced within biofilms, the levels may still be high enough for resistance selection to occur, at least in the early phases of biofilm formation when cells are dividing actively.

## MATERIALS AND METHODS

### Strains, media, and growth conditions.

All strains used in this study are listed in Table S1 in the supplemental material. These strains are all derived from Escherichia coli CFT073 (DA47111), a uropathogenic E. coli (UPEC) strain previously isolated from urine and blood samples of a patient with acute pyelonephritis (38). The use of chromosomal copies of an orange (*dTomato*) or a yellow (*SYFP2*) fluorescent protein (50) allowed measurements of cell-to-cell ratios during the competition experiments; therefore, all competing antibiotic-resistant strains were constructed in two isogenic strains, DA56709 (*SYFP2*) and DA56711 (*dTomato*). All constructed strains were whole-genome sequenced to confirm the absence of any additional unwanted mutations. Brain heart infusion (BHI) broth (Oxoid Limited, UK) or lysogeny broth (LB) with no salt was used for liquid cultures, and LB agar (LA) (Sigma-Aldrich, USA) was used for growth on plates. Strains were grown at 37°C unless otherwise noted, with planktonic growth in plastic tubes with shaking (180 rpm) and static biofilm growth in plastic 96-well plates with lids.

10.1128/mbio.01447-22.5 TABLE S1 Escherichia coli strains used. Download Table S1, XLSX file, 0.01 MB. Copyright © 2022 Hjort et al. 2022 Hjort et al. https://creativecommons.org/licenses/by/4.0/ This content is distributed under the terms of the Creative Commons Attribution 4.0 International license.

### Strain construction.

All mutant strains were constructed with the λ red recombineering system using the pSIM5-cam (chloramphenicol) vector (DA50218). The strains were grown overnight with 12.5 mg/L chloramphenicol at 30°C, diluted 1:200 in no-salt LB complemented with 12.5 mg/L chloramphenicol, and grown with shaking (150 rpm) at 30°C to an optical density at 600 nm (OD_600_) of 0.20. When the cultures reached the target optical density, the cultures were transferred to a shaking 42°C water bath, inducing the expression of the temperature-controlled λ red genes. After 30 min, the cultures were placed on ice and washed three times with 10% glycerol. After the final centrifugation step, the cell pellets were resuspended in glycerol and mixed with DNA in Eppendorf tubes. Electroporation was performed in 50 μL of cell-DNA mix in 1-mm-gap electroporation cuvettes with a GenePulser Xcell system (Bio-Rad) at 1.8 kV, 2 μF, and 200 Ω. The transformants were recovered in no-salt LB with 12.5 mg/L chloramphenicol overnight at 30°C and spread onto plates containing the relevant antibiotics. All primers used are listed in Table S2 in the supplemental material.

10.1128/mbio.01447-22.6 TABLE S2 Primers used. Download Table S2, DOCX file, 0.02 MB. Copyright © 2022 Hjort et al. 2022 Hjort et al. https://creativecommons.org/licenses/by/4.0/ This content is distributed under the terms of the Creative Commons Attribution 4.0 International license.

### Biofilm growth and extraction.

To grow the biofilm, an in-house-developed biofilm growth system, FlexiPeg, was used (37). The FlexiPegs were printed using high-temperature resin (High Temp; Formlabs) at U-PRINT, Uppsala University’s three-dimensional (3D) printing facility at the Disciplinary Domain of Medicine and Pharmacy, using Formlabs form 3 (low-force stereolithography) 3D printers. The equipment is designed as a lid with removable pegs that fit over a 96-well flat-bottom plate (Thermo Scientific). By inoculating the wells, a biofilm can form on the FlexiPegs, where the lid allows the pegs to be moved between plates for cycling and washing purposes. Inoculated FlexiPegs were grown statically at 37°C in plastic containers with lids.

For biofilm extraction, the FlexiPegs were first washed by immersing them in 250 μL 1× phosphate-buffered saline (PBS) three times for 1 min each, with PBS replaced between dips. The design of the lid allows the FlexiPegs to be easily moved between the inoculated 96-well plate and a new plate with wells filled with PBS. When washed, the lid is placed into a holder above a rack specially designed to fit 24 glass tubes. The holder is positioned so that the FlexiPegs can be pushed through the lid from above, down into the glass tubes filled with 600 μL 1× PBS. To harvest the biofilm from the FlexiPegs, the tubes were vortexed at high speed for 2 min.

### Competition experiments in a biofilm.

The introduction of either a yellow (*SYFP2* [CH2037]) or an orange (*dTomato* [CH6016]) fluorescent gene in the otherwise isogenic resistant mutant strains and the corresponding susceptible wild type allowed ratio determinations during the competition assays (Fig. 1). Cultures of the wild-type strain grown overnight in BHI medium were mixed 1:1 (unless otherwise noted) with a resistant mutant carrying the other of the two fluorescent markers and diluted 10,000-fold, and 200 μL was then transferred to each well, with a final concentration of 2 × 10^4^ to 6 × 10^4^ cells/well. The biofilm was then allowed to establish attachment on the FlexiPegs for 3 h at 37°C without the antibiotics, followed by medium changes 4, 5, 6, 7, 8, 9, and 10 h after the inoculum with added antibiotics until a total of 12 h of growth from inoculation. Between each plate shift, the FlexiPegs were submerged in 250 μL fresh BHI broth. For each antibiotic concentration, competition was performed with three unique biological replicates, with a dye swap, resulting in six replicates per data point. All FlexiPegs were then harvested as described above, except for one set of three FlexiPegs that was harvested after 3 h, before the onset of selection pressure. For the harvested biofilms, the ratios between the strains were determined using the MACSQuant VYB device (Miltenyi Biotec), counting 10^5^ events per sample. The samples were prepared for analysis by diluting 20 μL of the culture in 200 μL of 1× PBS in 96-well plates (Thermo Scientific).

### Competition experiments during planktonic growth.

The competition experiments during planktonic growth were performed using the fluorescent markers in the same way as described above, mixing a susceptible wild-type strain with a resistant mutant carrying different fluorescent tags. Cultures grown overnight in BHI broth were mixed 1:1 (unless otherwise noted) and diluted 1,000-fold in 1 mL medium containing a range of antibiotic concentrations below the MIC. For each antibiotic concentration, competition was done with 8 biological replicates, with a dye swap, resulting in 16 replicates per data point and experiment. The cultures were then grown under shaking conditions for 24 h at 37°C for 10 generations of growth. One microliter of each culture was then added to fresh BHI broth (1 mL) with the selected antibiotic concentration and grown for an additional 24 h. Cycling was performed two times, resulting in a total of 30 generations of growth.

After every 24 h of growth, the ratios between the competing strains were determined for each antibiotic concentration in all replicates, providing 16 independent ratio measurements per antibiotic concentration at 10, 20, and 30 generations. From all cultures, 2 μL was mixed with 200 μL 1× PBS in 96-well plates (Thermo Scientific) and measured using the MACSQuant VYB device (Miltenyi Biotec), counting 10^5^ events per sample.

### Fitness cost and MSC calculations.

To calculate the fitness cost and MSC values for the antibiotics, the selection coefficients were determined using the regression model *s* = ln[*R*_(_*_t_*_)_/*R*_(0)_]/(*t*), where *R* is the ratio of the resistant mutant to the susceptible wild type obtained by MACSQuant analysis. By plotting the *s* values as a function of the antibiotic concentration, the intercept on the *y* axis represents the fitness cost, and the intercept on the *x* axis equals the MSC value (12). The *s* values as a function of the antibiotic concentration for each biological replicate, including a dye swap, were plotted separately (16 biological replicates for planktonic growth and 6 for biofilm growth, with the exception of fosfomycin, which had only 4 biological replicates). The fitness costs and MSCs were calculated for each biological replicate with the standard errors of the means for each antibiotic and growth (biofilm and planktonic growth). For MSC calculations, biological replicates missing an MSC value due to the lack of a fitness cost were excluded from the calculation of the average MSC value and the error of the mean (in the trimethoprim biofilm experiment, one out of six replicates in biofilms showed no fitness cost and was excluded). The *P* value was calculated by a two-tailed *t* test with Welch’s corrections.

### Minimal biofilm inhibitory concentration.

In biofilms, the minimal biofilm inhibitory concentration (MBIC) can be used to measure bacterial tolerance toward antibiotics. The MBIC measures the impact of antibiotic treatment on a preformed biofilm, in our case after 24 h. For the determination of MBICs, cultures of the susceptible wild type grown overnight were diluted, and 200 μL/well was inoculated with 6 × 10^4^ cells/well. The MBIC was determined after 24 h, meaning that the biofilms were allowed to establish for 24 h without selection pressure, after which antibiotics were added. As for the competition experiments, a range of antibiotic concentrations was tested. After 24 h, the FlexiPegs were moved to medium containing the antibiotic, grown for an additional 24 h, and then harvested. After dilution and plating of the harvested biofilm, the CFU per FlexiPeg were determined and plotted against the antibiotic concentration. The MBIC was determined as the antibiotic concentration where fewer than 200 CFU were detected in the biofilm extracted from each FlexiPeg.

### MIC measurements.

MIC assays were performed by broth microdilution in round-bottom 96-well plates (Thermo Scientific) according to EUCAST guidelines. A few colonies were diluted in 1 mL 0.9% NaCl to a final OD_600_ of a 0.5 McFarland standard. One hundred microliters of the bacterial suspension was diluted in 10 mL of medium, giving a concentration of 1 × 10^8^ CFU/mL. Fifty microliters of the suspension was added to wells prefilled with 50 μL of medium with decreasing concentrations of the relevant antibiotic. The final CFU per well were 0.5 × 10^6^ to 1 × 10^6^ CFU per mL. The MIC was determined after static growth at 37°C for 16 to 20 h by identifying the well with the lowest concentration of the antibiotic without visible growth. In the case of pinpointing or skipped wells, EUCAST guidelines were followed when reading the results.

### PCR and local sequencing.

PCR amplification of mutated regions was performed using DreamTaq green PCR master mix (2×) (Thermo Scientific) with primers binding upstream and downstream of the mutation (see Table S2 in the supplemental material). The GeneJet gel extraction kit (Thermo Scientific) was used for purification, and the product was sent to Eurofins Genomics Europe for sequencing.
