# Supplementary material for: Antibiotic Minimal Selective Concentrations and Fitness Costs during Biofilm and Planktonic Growth
Source: mBio. 2022 Jun 13;13(3):e01447-22. doi: 10.1128/mbio.01447-22 (PMC9239065; doi:10.1128/mbio.01447-22)
Supplement: TABLE S2 [file mbio.01447-22-s0006.docx]

| Oligo/Primer | Sequence (5´to 3´) | Description |
| --- | --- | --- |
| uhpT stop | TTCGAGCGGAAGGTCCA  GGGTCGGCTTGCGAACCT  GGTT*TTA*GAAAGCCAGCA  TGGGTTACTCCTGAAATGA  ATACCTGC | Oligo for construction of the *uhpT* stop 5 aa mutant, fosfomycin resistance |
| uhpT-F | TGGCCCGCAGATGTTAAT | Forward PCR and sequencing primer for *uhpT* |
| uhpT-R | TGGCAGACAGGATCAGCA | Reverse PCR and sequencing primer for *uhpT* |
| △nfsA | TGTTAATAATCGCCTCACGCT  GCGCTTCGGAAATGGGTTAA  TCAGTGAAATGGCGAATGGA  GCGATGGCCACAAATAAGT | Oligo for construction of the *△nfsA* mutation, nitrofurantoin resistance |
| nfsA-F | ACCCGGACGACCAAAAAT | Forward PCR and sequencing primer for *nfsA* |
| nfsA-R | AAGGCACAGCCCAAACAG | Reverse PCR and sequencing primer for *nfsA* |
| △nfsB | TTTCACATGGAGTCTTTATGG  ATATCATTTCTGTCGAGCGTC  ATTCCACTAAGGCATTTGATG  CCAGCAAAAAA | Oligo for construction of the *△nfsB* mutation, nitrofurantoin resistance |
| nfsB-F | GCGAGGCATCAAGCATTT | Forward PCR and sequencing primer for *nfsB* |
| nfsB-R | GAAACGCCTGGCTCTTGA | Reverse PCR and sequencing primer for *nfsB* |
| rpsL K42N | CGTGGCGTATGTACTCGTGT  ATATACTACCACTCCT*AAC*A  AACCGAACTCCGCGCTGCGT  AAAGTATG CCGTGTTCGTCT | Oligo for construction of the *rpsL* K42N mutant, streptomycin resistance (*K42N*) |
| rpsL K42R | CGTGGCGTATGTACTCGTGTA  TATACTACCACTCCT*CGA*AAAC  CGAACTCCGCGCTGCGTAAAGT  ATGCCGTGTTCGTCT | Oligo for construction of the *rpsL* K42R mutant, streptomycin resistance (*K42R*) |
| rpsL-F | ACTTGGAACGAGCCTGCTTA | Forward PCR and sequencing primer for *rpsL* |
| rpsL-R | CTGCTCAGTGAAGGTGACGA | Reverse PCR and sequencing primer for *rpsL* |
| rpoB S531L | GCCTGCACGTTCACGGGTCAGA CCGCCTGGGCCAAGTGC*CAA*GAT ACGACGTTTGTGCGTAATCTCAG ACAGCGGGTTGT | Oligo for construction of the *rpoB* S531L mutation, rifampicin resistance (*S531L*) |
| rpoB-F | CCTGAGCAAAGACGACATCA | Forward PCR and sequencing primer for *rpoB* |
| rpoB-R | TACCTGCTTCACCCGGATAC | Reverse PCR and sequencing primer for *rpoB* |
| dfr-gene-F | ATGTTAACGGTGTAGCTATCA  GAATACGGAAGGCGCGAAGTT  CACCAGACAAATCCCAAT | Forward oligo for amplification of *dhfr* from DA68032 |
| dfr-gene-R | ACAACGCGCTCGTTGCACTTTC  GATCCCTCTGATTATTCGAATA  TCCCTTTATGGTGCAAAG | Reverse oligo for amplification of *dhfr* from DA68032 |
| dfr-F | CCACAATTGACTGCAACGTC | Forward PCR and sequencing primer for *dhfr* |
| dfr-R | CGACTCAGGATTACGCAAAA | Reverse PCR and sequencing primer for *dhfr* |
